# Value of Magnifying Chromoendoscopy and Magnifying Optical Enhancement Technology in Classifying Colorectal Polyps: A Prospective Controlled Study

**DOI:** 10.1155/2021/5533657

**Published:** 2021-08-27

**Authors:** Ying-Hao Song, Ruo-Xin Xu, Yong Zhang, Meng-Xuan Xing, Li-Dong Xu, Kun-Kun Li, Xing-Guo Xiao, Lu Li, Yan-Jing Xiao, Yu-Lei Qu, Ying-Jie Ma, Bao-Hui Jia, Hui-Li Wu

**Affiliations:** ^1^Department of Gastroenterology, People's Hospital of Henan University of Chinese Medicine, Henan University of Chinese Medicine, Zhengzhou, 450000 Henan Province, China; ^2^Department of Gastroenterology, Zhengzhou Central Hospital Affiliated to Zhengzhou University, Zhengzhou University, Zhengzhou, 450000 Henan Province, China; ^3^Jiangxi Medical College, Nanchang University, Nanchang, 330006 Jiangxi Province, China; ^4^Xinxiang Medical University, Xinxiang, 453000 Henan Province, China; ^5^Department of Pathology, Zhengzhou Central Hospital Affiliated to Zhengzhou University, Zhengzhou University, Zhengzhou, 450000 Henan Province, China; ^6^Department of Critical Care Medicine, The Fourth Affiliated Hospital of Nanchang University, Nanchang University, Nanchang, 330000 Jiangxi Province, China

## Abstract

**Background and Aims:**

Magnifying chromoendoscopy (ME-CE) through the observation of pit patterns is a productive way to distinguish between neoplastic and nonneoplastic polyps. Magnifying optical enhancement technology (ME-OE) is an emerging virtual chromoendoscopy imaging technology and appeared to be a promising approach. However, this information is currently not available. This study is aimed at comparing the differential diagnostic value of ME-CE and OE for neoplastic and nonneoplastic polyps. *Patients and Methods*. Consecutive patients undergoing colonoscopy were randomized (1 : 1) into examination by ME-OE or ME-CE. Histopathological findings were utilized as the reference standard. Accuracy, sensitivity, specificity, and positive and negative predictive values of two endoscopy methods were compared using ME-OE (were classified according to the JNET classification) and ME-CE (were classified according to the Kudo pit pattern classification), respectively, and the time to predict the histological polyp type was compared. And the agreements between the pathological and clinical diagnosis by ME-OE or ME-CE were analyzed.

**Results:**

A total of 365 polyps were found in the 220 patients included (ME-OE: 185; ME-CE: 180.202 had nonneoplastic polyps, 163 had neoplastic polyps). The diagnostic accuracy of ME-OE was higher than that of ME-CE (93% vs. 92%, *p* > 0.05). The average diagnosis time was lower in ME-OE than ME-CE (83 ± 26.4 s vs. 194 ± 17.7 s, *p* < 0.001). The agreements between the pathological and clinical diagnosis were at least substantial in both groups.

**Conclusion:**

ME-OE was superlative to ME-CE in predicting the histology of polyps. OE devoted classification would possibly similarly enhance the endoscopist performance. The trial is registered with ChiCT2000032075.

## 1. Introduction

In 2018, an additional 1.8 million new cases of colorectal cancer were diagnosed, with 881,000 deaths. Colorectal cancer accounts for one in 10 cancer cases and deaths, the 3rd incidence and the 2nd mortality [1, 2]. Although the incidence in China is lower than the world average, the number of new cases and deaths in China is the highest in the world. The adenoma-carcinoma series is the classic colorectal cancer (CRC) development paradigm, in which CRC starts as an adenoma. The endoscopic resection that follows will stop the condition from spreading and may also be a solution for intramucosal adenocarcinoma [3]. However, white light (WLE) colonoscopy alone is insufficient to distinguish neoplastic from nonneoplastic polyps, possibly resulting in removing a significant number of lesions that were not necessary [4]. Multiple endoscopic modalities have been recorded to be beneficial for colorectal polyp assessment [5].

Furthermore, since diminutive polyps account for the majority of polyps found during the colonoscopy, the ability to predict polyp histology in real time is clinically significant [6]. The JNET (The Japan NBI Expert Team) classification consists of four categories, types 1, 2A, 2B, and 3, based on vessel and surface pattern findings. The morphological appearance of each JNET type is then correlated with the histology, from benign hyperplastic polyps to advanced carcinomas [7].

Since the 1970s, Japanese endoscopists have used chromoendoscopy, and the Kudo proposed pit pattern classification has been generally adopted. Using the Kudo pit pattern classification system, chromoendoscopy with or without magnification has been used to discern neoplastic from nonneoplastic polyps [8]. The optical enhancement (OE, Pentax Medical, Tokyo, Japan) technology was developed as a new system for visualizing the morphology of mucosal surface patterns. The early i-Scan system is utilized as it uses white light as a source of brightening. However, it does not fully meet current needs. OE is a novel technique of electronic chromoendoscopy. The innovated optical filters may achieve higher overall transmittance by connecting the peaks of the hemoglobin absorption spectrum (415 nm, 540 nm, and 570 nm), generating a continuous wavelength spectrum. There are two modes with different OE filters (mode 1 and mode 2) [9]. At present, there are only a few studies in the esophagus or stomach for OE [10–26]. It is appropriate to choose mode 1 because of the similar principle to NBI (narrow band imaging) [10–26]. Compared with NBI, there are only a few studies on classifying colorectal polyps currently available. Therefore, in this study, we evaluated and compared the detection efficacies of ME-OE and ME-CE for classifying colorectal polyps.

## 2. Method

This was a prospective, randomized, and single-center study conducted at a teaching hospital in Zhengzhou, China. All participating patients provided written informed consent. The Ethics Committee approved Zhengzhou Central Hospital Affiliated to Zhengzhou University's research protocol (202032), and the study is registered in the China Clinical Trial Registry (ChiCT2000032075).

### 2.1. Patients

Patients were randomly distributed to two blocks in a 1 : 1 ratio into the ME-OE or ME-CE groups and received indication (screening, symptoms, surveillance, and physical examination) assessment. The exclusion criteria were as follows: inadequate bowel preparation defined as a total BBPS score < 6 [27], previous colon resection, coagulation disorders, melanosis coli, patients with a family history of adenomatous polyposis, patient refusal, and pathology reports with no definitive diagnosis in polyps or incomplete colonoscopy.

### 2.2. Study Design

All procedures were performed with an EPK-i7000 processor (Pentax, Japan), high-definition endoscopes with magnification (EC-3890FZi), and a black silicone elastomer cap on the distal end of the colonoscopy (Pentax DiStal Rubber Hood OE-A59, Pentax, Tokyo, Japan). All patients received a low-residue diet two days before the colonoscopy, oral polyethylene glycol cathartic agent 3L, to make the bowel preparation meet the requirements. All patients were given nasal cannula oxygen during the anesthesia process, a certified anesthetist performed the entire anesthesia process, and vital signs were monitored throughout the procedure. In our study, antispastic agents were not provided.

**Figure 1:**
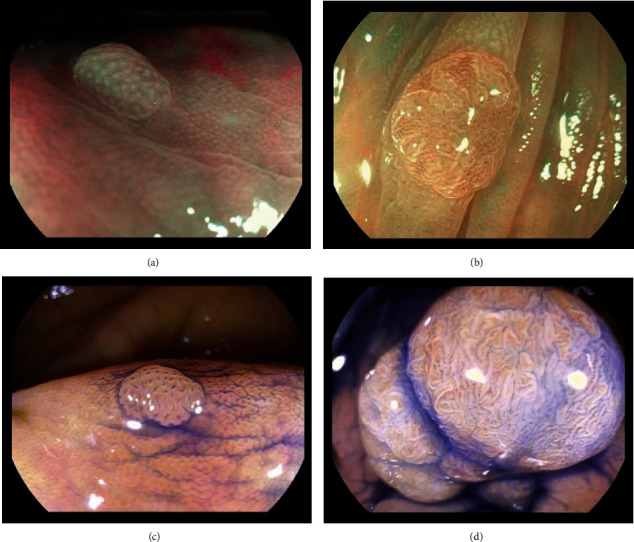
Endoscopic images in the magnifying mode: (a) nonneoplastic polyps (JNET type 1); (b) neoplastic polyps (JNET type 2A); (c) nonneoplastic polyps (pit pattern types I); (d) neoplastic polyps (pit pattern type IV).

A complete examination was defined as reaching the caecum using WLE. The effectiveness of the bowel cleansing was assessed according to the Boston Bowel Preparation Scale (BBPS) at the withdrawal phase. When a macroscopically visible lesion was suspected by white light endoscopy, the polyps' location sizes and morphology were also recorded. To expose the microstructure and microvasculature on the surface of the polyp, pronase and simethicone were applied. The diagnosis of polyp histology was based on diagnostic criteria defined by JNET or Kudo classification. JNET type 1 was classified as nonneoplastic polyps; other types were considered neoplastic polyps [28]; pit patterns I and II were defined as nonneoplastic polyps, and pit pattern types III–V were defined as neoplastic polyp (Figure 1) [2]. We excluded SSA/P in this study.

**Figure 2:**
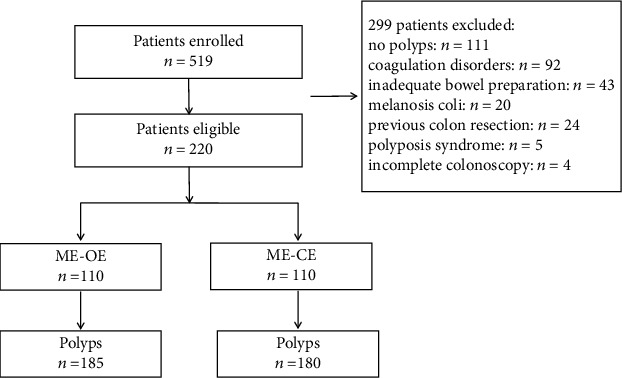
Flow chart of the examinations: 519 patients enrolled, 220 patients eligible (299 patients excluded: no polyps: *n* = 111, coagulation disorders: *n* = 92, inadequate bowel preparation: *n* = 43, melanosis coli: *n* = 20, previous colon resection: *n* = 24, polyposis syndrome: *n* = 5, and incomplete colonoscopy: *n* = 4).

The study flowchart is shown in Figure 2. The overall study was categorized as stages 1 and 2, and we considered that if the number of polyps in two groups at different stages in the same period of time might be biased, we chose the median number of polyps in two groups as the node of two stages. All experiments were performed by an endoscopist, who had extensive experience with the use of NBI (more than 5 years) and was familiar with the JNET or Kudo classification and also received 2 weeks of specialized endoscopic training. Patients were randomized to the ME-OE, and OE mode 1 was turned on. After the surfaces were zoomed in to observe microstructure and microvasculature, the prediction was reported to the assistant. The assistant recorded the time from turning on mode 1 to reaching a diagnosis. In patients randomized to the ME-CE, a total of 20 mL of 0.3% indigo carmine dye was evenly sprayed on the surface of polyps using a spray tube, and excess solution was aspirated after the spraying, making a magnified observation, and the prediction was reported to the assistant. The assistant recorded the time from inserting the spray tube to reaching the diagnosis and withdrawing the spray tube. During the whole process, any communication forms between the endoscopists were not allowed. The location was estimated by the anatomic landmarks. The size was evaluated by comparison with the span of open biopsy forceps. Polyp morphology classification was described by the Paris Classification [7]. Polyps were resected en bloc (by using forceps for <3 mm polyps or snare, cold or hot as appropriate, for larger ones) [7].

### 2.3. Pathological Polyp Evaluation

The tissue specimens collected during endoscopies were placed into formalin solution for 24 hours, subjected to conventional dehydration, paraffin embedding, sectioning, and then staining using the hematoxylin-eosin (H/E) staining method. Two experienced pathologists reviewed the slides independently and reached histological conclusions without knowing the endoscopic findings. If the results of the two pathologists were notably different, a third pathologist was consulted. The diagnostic histopathological criteria were based on the Vienna classification [10].

### 2.4. Outcome Measures

With histopathological evaluation as a reference standard, we evaluated the accuracy, sensitivity, specificity, and positive and negative predictive values of ME-OE and ME-CE endoscopy for polyp histology prediction, as primary outcomes.

The secondary outcome measures included comparing the time needed to predict polyp histology and the agreements between the pathological and clinical diagnosis by ME-OE or ME-CE.

### 2.5. Sample Size Estimation

The overall diagnostic accuracy in differentiating neoplastic from nonneoplastic lesions in their series was 80% [11]. We have considered clinically relevant an absolute difference of 15% [7]. All statistical tests were two-sided with a significance level of 5%, and statistical power, 1-*β*, is set to be 80%. With 10% expected dropouts, the total sample size was 220, who prudentially assume a detection of only one polyp. Combined with my endoscopic center, the detection rate of polyps is about 35%. We planned to enroll 628 patients.

### 2.6. Statistical Analysis

Statistical analysis was performed using SPSS 26.00 statistical software. Frequencies with percentages were used to represent qualitative variables, whereas means and standard deviations were used to describe quantitative variables. Comparisons of qualitative variables were conducted using Fisher's exact probability test or chi-squared test, while for comparisons of continuous variables, we employed a *t*-test. Consistency analysis was evaluated by the kappa test. The larger the kappa, the higher the agreement.

## 3. Results

### 3.1. Patients and Polyps

From August 2019 to May 2020, we screened 519 patients, and after, 229 patients were excluded (Figure 2). A total of 220 patients were included (ME-OE: 185 polyps in 110, ME-CE: 180 polyps in 110). There were similarly no differences in age, sex, bowel preparation, or indications for colonoscopy between the two groups' (Table 1) accessibility, size, morphology, anatomical location, and pathologic diagnosis (Table 2).

### 3.2. Primary End-Point

Our main targets here are to compare the accuracy of polyp diagnosis between groups.

The overall accuracies were 93% vs. 92% for ME-OE and ME-CE, respectively, *p* > 0.05. The accuracy of ME-OE in stage 1 and 2 was 90% vs. 96%, *p* > 0.05. The accuracy of ME-CE in stages 1 and 2 was 90% vs. 93%, *p* > 0.05 (Table 3). In stage 1, the overall accuracies were 90% and 90% for ME-OE and ME-CE, *p* > 0.05. In stage 2, the overall accuracies were 96% for ME-OE and 93% for ME-CE, *p* > 0.05 (Table 4).

### 3.3. Secondary End-Points

In addition to our objectives of primary interest, we examined the following secondary purposes. The average diagnosis time between the two groups and various stages in the same groups was compared: 83 s ± 26.4 s for ME-OE and 194 s ± 17.7 s for ME-CE (*p* < 0.001), 96 s ± 26.8 s and 70 s ± 19.0 s (*p* < 0.001) in two stages of ME-OE, and 20.5 s ± 13.9 s and 183 s ± 14.0 s (*p* < 0.001) in two stages of ME-CE (Table 3).

The average diagnosis time between the various groups in the same stages was compared: 96 s ± 26.8 s for ME-OE and 205 s ± 13.9 s for ME-CE (*p* < 0.001) in stage 1 and 70 s ± 19.0 s for ME-OE and 183 s ± 14.0 s for ME-CE (*p* < 0.001) in stage 2 (Table 4).

The agreements between the pathological and clinical diagnosis between the two groups and various stages in the same groups were compared: both almost perfect for ME-OE (*k* = 0.859) and ME-CE (*k* = 0.827), both almost perfect (*k* = 0.804 and 0.912) in 2 stages of ME-OE, and substantial (*k* = 0.800) for stage 1 and nearly perfect (*k* = 0.848) for stage 2 in ME-CE (Table 3).

## 4. Discussion

Three aspects were evaluated in our prospective randomized study. First, the diagnostic accuracy of ME-OE was higher than that of ME-CE. Second, the average diagnosis time was lower in ME-OE than in ME-CE. Third, the agreements between the pathological and clinical diagnosis at least were substantial in both groups.

In older studies, the accuracy of polyps categorized as nonneoplastic or neoplastic lesions by conventional endoscopy was shown to be 80% by the Kudo classification [16]. In the subsequent research, Calderwood et al. showed that ME-CE could distinguish between neoplastic and nonneoplastic polyps with an accuracy of 80.1% [15]; Hirata et al. reported that differentiation between neoplastic and nonneoplastic lesions was possible with a 92% sensitivity and a 73.3% specificity [17]. Overall, the diagnostic accuracy in differentiating neoplastic from nonneoplastic lesions was 88.4%. In meta-analysis [18], the Kudo classification has great significance in the identification of colorectal neoplasm. Tung et al. showed that ME-CE is a reliable tool to predict and differentiate between neoplastic and nonneoplastic polyps [20]. Optical enhancement (OE) was recently developed by PENTAX, and few studies have been conducted in this field. However, several studies suggest that the JNET classification can provide immediate histological diagnosis and more reliable estimation of the depth of invasion by magnifying endoscopy [19–23].

Compared to the accuracy of the two groups, the diagnostic accuracy of ME-OE was significantly higher than that of ME-CE (93% vs. 92%, *p* > 0.05). In stage 1, the overall accuracies were 90% and 90% for ME-OE and ME-CE, *p* > 0.05. In stage 2, the overall accuracies were 96% for ME-OE and 93% for ME-CE, *p* > 0.05. Whether from the overall accuracy or the same group of different stages, the same stage of the other groups' accuracy is more than 90% accurate. The agreements between the pathological and clinical diagnosis are healthy at all times. It can be seen that both groups have effective methods to diagnose the nature of polyps. This point needs attention. The performance of ME-OE in classifying colorectal polyps is much better than ME-CE at all times. Especially in OE stage two, the specificity was 100%, and there were no cases with misdiagnosis. While the ME-CE is also excellent, the ME-OE seems to be the better choice.

In terms of diagnosis time, ME-OE was also faster and convenient than ME-AAC (83 s ± 26.4 s vs. 194 s ± 17.7 s). This superiority continues until the time point before the last. This may be related to the operation process of pigment endoscopy, which requires not only delivering the spray tube to the intestinal cavity and fixing the spray tube to a specific position to spray accurately but also removing the excess solution. Besides, there are differences between different stages within the same group. In both groups, the second stage was faster than the first. This might be due to a lack of experience in the early. In a study of endoscopists with different experiences, after 20 minutes of NBI teaching [17], 37 doctors' accuracy in distinguishing adenomas from proliferative polyps rose from 47.6% to 90.8%, suggesting that short education can improve doctors' judgment on the nature of polyps. In our study, no matter in the OE group or the CE group, after the first stage, the accuracy of judging the nature of polyps by endoscopists in the second stage has been significantly improved, which seems to show that practical learning plays an essential role in enhancing the ability of doctors. According to the European Society of Gastrointestinal Endoscopy (ESGE) [17, 18], it is significant for optical diagnostics by self-directed learning or practical courses. It is feasible that virtual chromoendoscopy and dye-based chromoendoscopy can be used for optical diagnosis of limited to diminutive polyps (≤5 mm) without the need for pathologic confirmation, but only if adequately photodocumented which would be required to support endoscopists' claims of adenoma detection, and also emphasized experienced endoscopists who are adequately trained.

This study also had some limitations. Namely, this was a single-center study with a small sample size, and thus, further in-depth, large-scale, and multicenter studies are required in the future. Besides, no specific classifying colorectal polyps for ME-OE are available thus far, and we hope that a ME-OE diagnostic standard can be established shortly. The examination was performed by experienced endoscopists, which may lead to selection bias. As the limitation of the experimental specimens, we did not collect all the polyp types, which may have an individual impact on the conclusion.

Overall, the study adds to our understanding of classifying colorectal polyps by ME-OE. And the ability of ME-OE was significantly better than that of ME-CE. The method not only has high accuracy but also is simple to perform with ME-CE and saves time. Furthermore, practical learning is necessary to raise diagnostic accuracy. In the future, we should further verify the ability to use OE to identify various polyp types based on JNET classification.

## Figures and Tables

**Table 1:** Clinical characteristics of the subject subjects.

	ME-OE	ME-CE	Overall	*p* value
Number	110	110	220	NA
Gender (M/F)	56/54	53/57	109/111	0.686
Age (mean ± SD) (y)	48.8 ± 14.1	51.7 ± 14.3	50.3 ± 14.2	0.121
Indication				0.154
Screening	21	14	35	
Symptoms	43	34	77	
Surveillance	23	35	58	
Examination	23	27	50	
Bowel cleansing (BBPS)				0.08
6/7	7/17	6/32	13/49	
8/9	47/39	34/38	81/77	

ME-CE: magnifying chromoendoscopy; ME-OE: magnifying optical enhancement technology; BBPS: Boston Bowel Preparation Scale; NA: not applicable; SD: standard deviation.

**Table 2:** Descriptive per-polyp analysis.

	ME-OE (%)	ME-CE (%)	Overall (%)	*p* value
Number	185	180	365	NA
Size (mm)				0.157
≤5	104 (56.2)	119 (66.1)	223 (61.1)	
5-10	76 (41.1)	58 (32.2)	134 (36.7)	
≥10	5 (2.7)	3 (1.7)	8 (2.2)	
Location				0.389
Right side of colon	73 (39.5)	64 (35.6)	137 (37.5)	
Transverse colon	35 (18.9)	26 (14.4)	61 (16.7)	
Descending colon	40 (21.6)	44 (24.4)	84 (23.1)	
Sigmoid colon/rectum	37 (20.0)	46 (25.6)	83 (22.7)	
Morphology				0.064
Ip	23 (12.5)	33 (18.3)	56 (15.3)	
Is	62 (33.5)	73 (40.6)	135 (37.0)	
IIa	77 (41.6)	61 (33.9)	138 (37.8)	
IIb	23 (12.4)	13 (7.2)	36 (9.9)	
Histology				0.120
Nonneoplastic	95 (51.4)	107 (59.4)	202 (55.3)	
Size (mean ± SD) (mm)	3.1 ± 1.1	3.3 ± 1.2	3.2 ± 1.1	
Neoplastic	90 (48.6)	73 (40.6)	163 (44.7)	
Size (mean ± SD) (mm)	5.9 ± 1.7	5.8 ± 1.4	5.8 ± 1.6	
Time (mean ± SD) (s)	83 ± 26.4	194 ± 17.7	138 ± 60.2	0.001

ME-CE: magnifying chromoendoscopy; ME-OE: magnifying optical enhancement technology; NA: not applicable; SD: standard deviation.

**Table 3:** Diagnostic accuracy and time in two methods.

	Overall *n* = 365		ME-OE *n* = 185		ME-CE *n* = 180	
	ME-OE *n* = 185	ME-CE *n* = 180	Stage 1 *n* = 92	Stage 2 *n* = 93	Stage 1 *n* = 90	Stage 2 *n* = 90
Sensitivity (%)	91	85	96	90	87	84
Specificity (%)	95	96	85	1	93	98
PPV (%)	94	94	86	1	93	96
NPV (%)	92	90	95	93	88	92
Accuracy (%)	93	92	90	96	90	93
*p* value	0.639		0.145		0.418	
*K*	0.859	0.827	0.804	0.912	0.800	0.848
Time (mean ± SD) (s)	83 ± 26.4	194 ± 17.7	96 ± 26.8	70 ± 19.0	205 ± 13.9	183 ± 14.0
*p* value	0.001		0.001		0.001	

PPV: positive predictive value; NPV: negative predictive value; ME-CE: magnifying chromoendoscopy; ME-OE: magnifying optical enhancement technology; SD: standard deviation.

**Table 4:** Diagnostic accuracy and time in two stages.

	Overall *n* = 365		Stage 1 *n* = 182		Stage 2 *n* = 183	
	ME-OE *n* = 185	ME-CE *n* = 180	ME-OE *n* = 92	ME-CE *n* = 90	ME-OE *n* = 93	ME-CE *n* = 90
Sensitivity (%)	91	85	96	87	0.90	84
Sensitivity (%)	95	96	85	93	1	98
PPV (%)	94	94	86	93	1	96
NPV (%)	92	90	95	88	93	92
Accuracy (%)	93	92	90	90	96	93
*p* value	0.639		0.961		0.705	
*K*	0.859	0.827	0.804	0.800	0.912	0.848
Times (mean ± SD) (s)	83 ± 26.4	194 ± 17.7	96 ± 26.8	205 ± 13.9	70 ± 19.0	183 ± 14.0
*p* value	0.001		0.001		0.001	

PPV: positive predictive value; NPV: negative predictive value; ME-CE: magnifying chromoendoscopy; ME-OE: magnifying optical enhancement technology; SD: standard deviation.

## Data Availability

No additional data are available.

## Additional Points

*Clinical Trial Registration Statement*. This study was registered at chictr.org.cn. The registration identification number is ChiCT2000032075.
